# Downregulation of transcription factor SOX2 in cancer stem cells suppresses growth and metastasis of lung cancer

**DOI:** 10.1038/bjc.2011.94

**Published:** 2011-04-05

**Authors:** R Xiang, D Liao, T Cheng, H Zhou, Q Shi, T S Chuang, D Markowitz, R A Reisfeld, Y Luo

**Affiliations:** 1 Department of Immunology and Microbial Science, The Scripps Research Institute, 10550 North Torrey Pines Road, La Jolla, CA 92037, USA; 2 Department of Immunology, Beijing Union Medical School, Beijing 100010, China; 3 Department of Immunology, Nankai University, Tianjin 300071, China

**Keywords:** cancer stem cells, side population, non-small cell lung tumour cells, transcription factor, SOX2, Hoechst 33342 dye

## Abstract

**Background:**

The cancer stem cell hypothesis suggests that neoplastic clones are maintained exclusively by a small subpopulation of cells, which have indefinite proliferation and differentiation potentials and give rise to phenotypically diverse cancer cells. Cancer stem cells have been isolated by their ability to efflux Hoechst 33342 dye and are referred to as the ‘side population’ (SP).

**Methods and results:**

The Hoechst efflux assay was used to isolate and characterize the SP from murine D121 lung carcinoma cells. Here, we demonstrated that D121-SP cells contain cancer stem cell characteristics, that is, upregulation of the transcription factors SOX2 and Oct 4 in D121-SP cells. In addition, the migration of D121-SP was decreased, and apoptosis of D121-SP was upregulated following knocking down of SOX2 in D121 cells. Importantly, downregulation of SOX2 in D121 cells markedly suppressed their metastatic potential in syngeneic mice.

**Conclusions:**

These results suggest that the SP is an enriched source of lung tumour cells with stem cell properties and that SOX2 has an important role in maintaining stem cell properties and functions that may be a potential target for effective lung cancer therapy.

The cancer stem cell hypothesis suggests that neoplastic clones are maintained exclusively by a small subpopulation of cells, which have an indefinite proliferation and differentiation potential and give rise to phenotypically diverse cancer cells (Reya *et al*, 2001; Pardal *et al*, 2003). The limited numbers of CSCs in the tumour cell population and their specific phenotypes are considered responsible for their escape from conventional cancer therapies resulting in disease relapse (Pardal *et al*, 2003). These CSCs were initially identified in haematologic tumours, such as leukaemia (Bonnet and Dick, 1997) and subsequently found in various solid malignancies such as brain, breast, prostate, and lung cancinoma (Singh *et al*, 1990; Al-Hajj *et al*, 2003; Kim *et al*, 2005; Xin *et al*, 2005). A useful approach for identification and purification of CSCs, specifically in the absence of suitable surface marker expression is based on the phenomenon that stem cells have the specific ability to efflux lipophilic, fluorescent dyes such as Hoechst 33342. The efflux of this dye was found to be correlated with ABC transporters, in particular ABCG2, (Zhou *et al*, 2001) which could be specifically inhibited by Ca^++^-channel blockers. This population of cells, low in Hoechst dye, was designated SP and was first observed in haematopoietic tumour where the bone marrow was greatly enriched in stem cells. (Spangrude and Johnson, 1990; Goodell *et al*, 1996). In addition, this dye efflux method was also applied to identify SP cells with stem cell properties in a variety of tissues such as mammary gland, skin, brain, and liver (Wulf *et al*, 2003; Kim and Morshead, 2003; Challen and Little, 2006).

In our previous studies, we successfully used this Hoechst 33342 dye method to isolate SP with CSC properties from murine 4T1 breast carcinoma and NXS2 neuroblastoma cell lines and transplanted them into the immune-competent microenvironment of syngeneic mice. These SP cells not only displayed characteristics of stem cell markers, but also exhibited increased resistance to chemotherapy when compared with non-SP cells. Most importantly, SP cells showed a markedly higher repopulation and tumourgenic potential *in vivo* (Kruger *et al*, 2006).

A number of links were recently found between SOX transcription factors and cancer (Dong *et al*, 2004). Thus, SOX2 was reported to be an immunogenic antigen in 41% of small cell lung cancer patients (Gure *et al*, 2000). Also, SOX2 was involved in later events of carcinogenesis such as invasion and metastasis of pancreatic (Sanada *et al*, 2006) and prostate (Sattler *et al*, 2000) cancers. In addition, SOX2 was expressed in 43% of basal cell like breast carcinomas (Rodriguez-Pinilla *et al*, 2007). Recently, molecular mechanisms governing the oncogenic potential of SOX2 in breast cancer were elucidated, indicating that this transcription factor promotes cell proliferation and tumourigenesis by facilitating the G1/S transition and through its transcriptional regulation of the *CCNDS* gene in breast cancer cells. In addition, *β*-catenin was identified as a transcription factor particular for SOX2 (Garcia-Lavandeira *et al*, 2009). Apparently, SOX proteins, including SOX2, bind to specific DNA sequences by means of their highly conserved high mobility group domain in functioning as transcription factors to activate or repress target gene expression (Kamachi *et al*, 2000; Wilson and Koopman, 2002). In fact, numerous transcription factors from a wide range of gene families were found to partner with SOX proteins (Kamachi *et al*, 2000; Wilson and Koopman, 2002) and such partnerships were found to be crucial in determining the functional specificity of SOX proteins. Another important factor, in this regard, is the identification of downstream targets of SOX proteins. Thus, transcriptional regulatory networks consist of functional interactions between regulatory genes and a much larger set of downstream target genes. The network of SOX2: Oct 4 target genes include among others SOX2, Oct 4, Nanog, FGF-4, UTF1, Fbx15 and Lefty1. Furthermore, each of these target genes is substantially downregulated upon differentiation of both embryonic stem cell (ESs) and embryonic cell, due to the downregulation of SOX2 and Oct 4 (Yuan *et al*, 1995; Chambers *et al*, 2003). Given the importance of SOX2/Oct 4 target genes during development, it is no surprise that their expression is tightly controlled. Thus, even small changes in Oct 4 levels can drastically alter cell fate. For example, a two-fold increase in Oct 4 levels caused ES cells to differentiate into endoderm and mesoderm, whereas knockdown of Oct 4 induced ES cells to differentiate into trophectoderm-like cells (Niwa *et al*, 2000). Clearly, the consequences of altering the level of Oct 4 have provided useful insights into the developmental potential of ES and EC cells. However, much less attention has been placed thus far on understanding how altering the levels of SOX2 influences cell fate. Although knockdown of SOX2 causes ES cells to differentiate (Chew *et al*, 2005; Rodda *et al*, 2005), there are few reports describing how elevating SOX2 levels influences the regulation of SOX2/Oct 4 target genes as well as generation of CSCs.

Here, we demonstrate that the CSC concept can also be applied to a mouse lung tumour model by identifying SP retaining low Hoechst 33342 dye in a murine D121 non-small cell lung carcinoma cell line. Importantly, this SP displays stem cell characteristics *in vitro*, which can be considerably enriched to highly express transcription factor SOX2. Importantly, siRNA-mediated knockdown of SOX2 in CSCs significantly suppresses tumour growth and experimental pulmonary metastases of D121 non-small cell lung tumour cells transplanted into syngeneic C57/BL6 mice.

## Materials and methods

### Animal cell lines and reagents

The C57BL/6 mice, 6–8 weeks of age, were purchased from The Jackson's Laboratory (Bar Harbor, ME, USA). All animal experiments were performed according to National Institutes of Health guidelines. The murine D121 non-small lung carcinoma cell line was generously provided by Dr Lea Eisenbach (Weizmann Institute of Science, Rehovot, Israel). The IFN-*γ* and MHC-I antibody were purchased from Invitrogen (Invitrogen, Carlsbad, CA, USA).

### Flow cytometry analysis and cell sorting

We stained 1 × 10^6^ tumour cells per ml with either 5 *μ*g ml^−1^ Hoechst 33342 dye (Sigma, St Louis, MO, USA) or Hoechst dye plus 100 mM (+/−) Verapamil hydrochloride (Sigma) and incubated such cells at 37°C for 25 min as described by us previously (Kruger *et al*, 2006). These cells were further stained with antibodies against Sca-1, c-Kit, ABCB1B or ABCG2 (BD Pharmingen, San Diego, CA, USA), and the appropriate isotype controls (BD Pharmingen). Then, 1 × 10^6^ viable cells were analysed and sorted by EPICSALTRA (Beckman Coulter, Fullerton, CA, USA). The Hoechst dye was excited at 350 nm, and its fluorescence measured at two wave length (450/20 nm band-pass filter and 675LP optical filter). Fluorescence-activated cell sorting (FACS) data were analysed with FlowJo software (Tree Star, Ashland, OR, USA).

### Reverse transcriptase (RT)–PCR analysis

Total RNA from three independently sorted D121-SP and non-SP cell samples was isolated using RNAeasy (Qiagen, Valencia, CA, USA). The RNA from D121 cells was transcribed in cDNA using the SuperScript First-Strand Synthesis System (Invitrogen). RT–PCR was performed with SuperScript One-Step (Invitrogen). Primers used were as follows: ABCG2 (sense, 5′-TCCTCCCAGACTTGGAAATG-3′ antisense, 5′-GGGTGTCTGTGGTAGGAGAA-3′), Sca-1 (sense, 5′-TGGACACTTCTCACACTACAAAGTC-3′ antisense, 5′-CAGAGCAAGGTCTGCAGGAG-3′), Wnt-1 (sense, 5′-ACCTGTTGACGGATTCCAAG-3′ antisense, 5′-GCCTCGTTGTTGTGAAGGTT-3′), Wnt-2 (sense, 5′-ATGAACGTCCCTCTCGGTGGAATCTGGCTC-3′ antisense, 5′-TCATGTAGGCGTCGCCCAGTCGGCACTCTT-3′), Wnt-10a (sense, 5′-GACTCCACAACAACCGTGTG-3′ antisense, 5′-TGGGGAAGGGAAGAAGAGAT-3′), SOX2 (sense, 5′-ACCAGCTCGCAGACCTACAT-3′ antisense, 5′-ATGGGCTCTGTGGTCAAGTC-3′), Oct 4 (sense, 5′-AGCTGCTGAAGCAGAAGAGG-3′ antisense, 5′-CCAATCAGCTTGGGCTAGAG-3′), Notch1 (sense, 5′-TTGACGTCACTCTCCTGTGC-3′ antisense, 5′-ACACAGGTGCCATTGTTGAA-3′), GAPDH (sense, 5′-CATTGACCTCAACTACATGG-3′ antisense, 5′-CACACCCATCACAAACATGG-3′).

### Immunohistochemical fluorescence staining

The D121 cultured tumour cells were initially stained with Hoechst 33342 dye for 25 min at 37°C. The cells were then washed twice with cold PBS and stained with rabbit anti-mouse SOX2 or Oct 4 mAb (ABD Serotec, Kidlington, UK) and goat anti-rat IgG Alexa 568 (Invitrogen) as the secondary reporter reagent. Rabbit anti-mouse Sca-1 or ABCG2 antibodies were purchased from Santa Cruz (Santa Cruz Biotechnology, Inc., Santa Cruz, CA, USA). Reactions were visualised with goat anti-rat IgG Alexa 488 (Invitrogen), and slides were analysed by fluorescence microscopy (Olympus, Munich, Germany).

### siRNA gene silencing

We used the siRNA gene Silencer system (Santa Cruz Biotechnology, Inc.) to perform the SOX2 gene silencing in D121 tumour cells by following the instructions provided by the manufacturer. The results of SOX2 expression in D121 were verified by RT–PCR.

### Migration assay

Transwell migration assays were performed with either SOX2 siRNA D121 or wild-type D121 tumour cells being harvested in modified Boyden chambers (Transwell; Corning Inc. Lowell, MA, USA). After 4 hr incubation at 37°C, the cells on the lower surface of wells were fixed with 1% paraformaldehyde, stained with 1% crystal violet, and counted.

### Apoptosis assay

For apoptosis studies, we used the Annexin V FCAS assay kit (eBioscience, San Diego, CA, USA) according to the manufacturer's protocol. Hoechst 33342 dye was added 25 min before incubation with Annexin V to identify the SP or non-SP cell populations. We also used the cleaved Caspas-3 antibody that was conjugated with Alexa Fluor 488 (Cell signaling technology Inc., Danvers, MA, USA) to stain with either SP or non-SP, which were detected with flow cytometry.

### *In vivo* tumour cell challenge

Groups of C57BL/6 mice (*n*=4) were injected i.v. with either 1 × 10^5^ D121 wild type or D121 SOX2 knockdown lung carcinoma cells. All mice were killed 25 days after tumour cell challenge.

### Statistical analysis

The statistical significance of different findings between experimental groups was determined by the Student's *t*-test. Results were considered significant if two-tailed *P*-values were <0.05.

## Results

### D121 non-small cell lung carcinoma cells contain a side population (SP) with cancer stem cell properties

**Figure 1:**
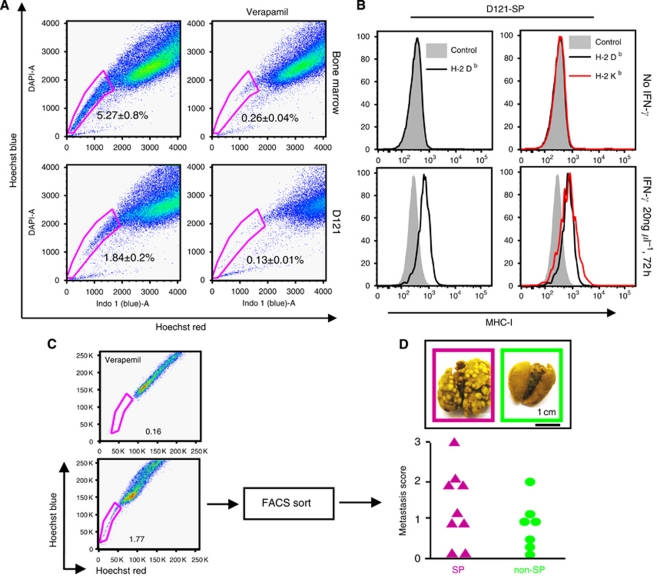
D121 non-small cell lung carcinoma cells contain a SP with cancer stem cell properties. (**A**) D121 lung cancer cells and mouse bone marrow cells were stained with Hoechst 33342 to determine a dye effluxing SP. (**B**) The MHC-I antigen expression on D121-SP cells was upregulated by incubation with INF-*γ*. (**C**) The SP of D121 cells was sorted by flow cytometry after being stained with Hoechst 33342 dye, and a group of C57/BL mice were implanted in the cleared fat pad with either the SP or non-SP fraction of D121 cancer cells at 1 × 10^5^ per mouse. (**D**) All mice were killed on day 25 after tumour cell implantation and analysed for the metastasis score on lungs.

To identify CSCs among D121 lung tumour cells, SP cells were isolated by staining with Hoechst 33342 dye and then subjected to flow cytometry with bone marrow cells serving as a positive control (Figure 1A, left). The SP was stained with Hoechst dye in the presence of the Ca^++^-channel blocker Verapamil hydrochloride to verify the specificity of the SP population (Figure 1A, right). FACS analysis of the sample clearly defined a SP population that constituted 1.84±0.2% within D121 tumour cells and 5.27±0.8% within the mouse bone marrow sample. Incubation with Verapamil significantly reduced the number of cells found within the SP to 0.13±0.01% in D121 tumour cells and to 0.26±0.04 in the bone marrow. These results suggest that D121 lung tumour cells contain SP of cells similar to that found within bone marrow. Moreover, to demonstrate the immunogenicity of SP cells, we cultured D121 cells with INF-*γ* (20 ng *μ*l^−1^ 72 h) and then stained with Hoechest 33342 to collect SP. Expression of MHC-class I antigen on SP cells was detected with H-2K^b^ or H-2D^b^ antibodies on SP from D121 cells and was found upregulated by incubation with INF-*γ*, providing an important potential target cell for immunotherapy (Figure 1B). Furthermore, we confirmed that such SP cells possess stem cell features *in vivo*. In this regard, groups of C57/BL mice (*n*=8) were implanted i.v. with either SP or D121 lung cancer cells (1 × 10^5^ per mouse) (Figure 1C). All mice were killed on day 25 after tumour cell implantation, and lung metastasis score was higher when applied to lungs of mice implanted with SP of D121 tumour cells than in mice implanted with non-SP control cells (Figure 1D). All data suggest that only the SP cells of D121 lung cancer cells contain the CSC fraction.

### Upregulation of stem cell markers on D121-SP

**Figure 2:**
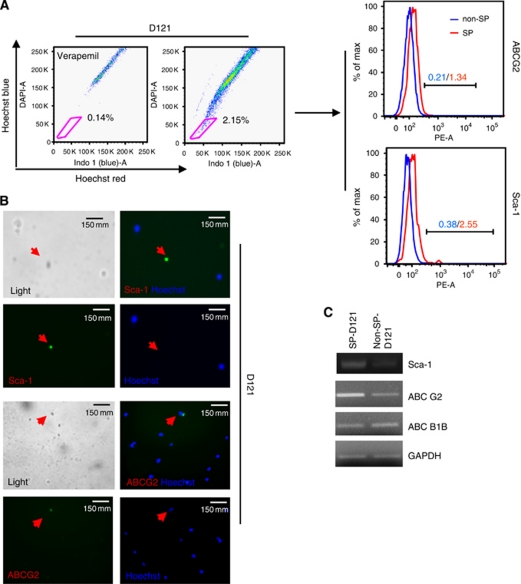
Upregulation of stem cell markers on D121-SP population. (**A**) Expressions of ABCG2 and Sca-1 stem cell markers were upregulated in the SP cells derived from D121 lung tumour cells as detected by flow cytometry, immunofluorescence histology staining (**B**) and RT–PCR (**C**).

To further establish the characteristics of SP cells among D121 tumour cells, we analysed for the expression of markers known to be associated with stem or progenitor cells. To this end, we performed flow cytometry, immunohistochemical fluorescence staining and RT–PCR, respectively, to verify the expression of Sca-1 and ABCG2 by different methods. We found expression of ABCG2 and Sca-1 to be upregulated in the SP cells isolated from D121 lung cancer cells when compared with that of non-SP derived from these same cancer cells as detected by flow cytometry (Figure 2A), by immunohistochemical fluorescence histology staining (Figure 2B) and RT–PCR (Figure 2C). Together, these results demonstrate that D121-SP cells express the stem cell marker Sca-1 as well as the ABCG2 ATP-binding cassette transporter, which is associated with resistance to multiple chemotherapeutic drugs. These results support our contention that D121-SP cells have a stem cell gene expression profile.

### D121-SP cells reveal upregulation of stem cell-associated transcription factors SOX2 and Oct 4

**Figure 3:**
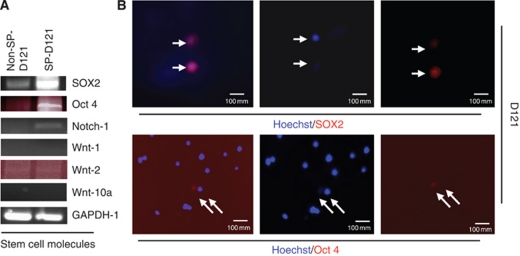
Upregulation of stem cell-associated transcription factors SOX2 and Oct 4 on D121-SP cells. (**A**) The expression of SOX2 and Oct 4, but not Wnt-1 and 10 were found to be upregulated in the SP cells derived from D121 lung cancer cells as detected by RT–PCR and immunoflorence histology staining (**B**).

To determine which key transcription factors are involved in maintaining stem cell characteristics we further analysed SP from D121 cells for the expression of these important stem cell-associated molecules. To this end, RT–PCR was performed to determine expressions of SOX2, Oct 4, Notch-1 and Wnt-1, 2 and Wnt-10a either in sorted SP or non-SP D121 cells. The results indicated that expressions of SOX2 and Oct 4, but not those of Wnt-1, 2 and10a, were upregulated in SP cells isolated from D121 tumour cells when compared with those of non-SP cells (Figure 3A). To further confirm these results, immunohistochemical fluorescence staining was used with either anti-SOX2 or Oct 4 antibodies (Figure 3B). Our findings clearly indicated a markedly higher expression of SOX2 and Oct 4 in D121-SP cells than that observed in non-SP cells. These data suggest that the transcription factor SOX2 together with its partner gene might have an important role in the maintenance of tumour stem cell characteristics.

### Downregulation of SOX2 gene expression results in inhibition of migration and increased apoptosis of D121 lung carcinoma cells

**Figure 4:**
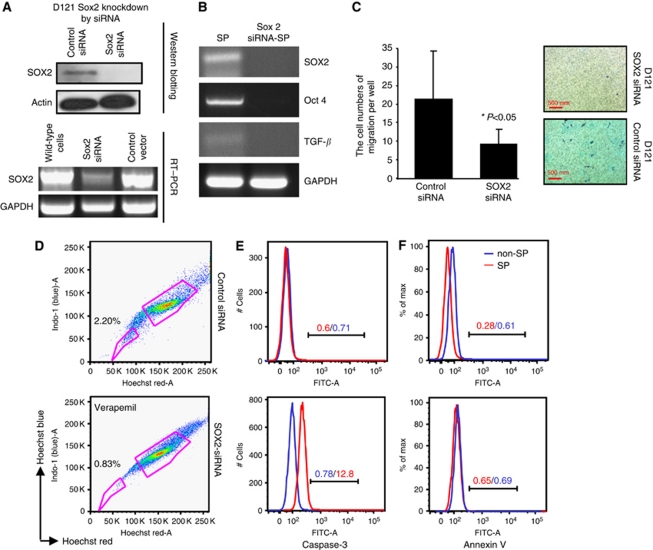
Inhibition of migration and increased apoptosis of D121 lung carcinoma cells by downregulation of SOX2 gene expression. (**A**) The expression of SOX2 in D121 lung cancer cells was downregulated by siRNA as verified by western blotting and RT–PCR. (**B**) Downregulation of TGF-*β* and Oct 4 while SOX2 expression of SOX2 was knocked down by siRNA. (**C**) Inhibition of D121 cells migration after downregulation of SOX2. (**D**–**F**) Increased apoptosis of D121-SP cells, which was indicated by increasing Caspase-3 and Annexin V after SOX2 knockdown in D121 cells.

To further explore SOX2 function in the SP of D121 tumour cells, we applied the siRNA technology to knockdown SOX2 gene expression. The expression of SOX2 at the RNA or protein level in D121 lung tumour cells was found to be markedly downregulated by siRNA as verified by western blotting and RT–PCR (Figure 4A). We also found that expressions of TGF-*β* and Oct 4 were downregulated when Sox2 was knocked down by siRNA (Figure 4B). Furthermore, when we knocked down SOX2 expression in D121 cells with siRNA and then performed the cell migration assay, we found a marked reduction of D121 cell migration (Figure 4C). More interesting, the percentages of D121-SP cells were markedly decreased after SOX2 downregulation (Figure 4D). In addition, when FACS analysis was used to detect apoptosis under these conditions, we observed a distinct increase in apoptosis of SP cells that was demonstrated by Annexin V and the expression of Caspase-3 in D121-SP cells (Figure 4E and F). These results suggest that SOX2 gene expression in the SP derived from D121 cancer cells could indeed have an important role in maintaining CSC characteristics, and regulate a target gene such as TGF-*β* that is able to control tumour progression and metastasis. Therefore, SOX2 could possibly serve as a potential target for improved treatment of lung cancer.

### SOX2 knockdown in D121 lung carcinoma cells suppressed experimental metastases in syngeneic C57/BL mice

**Figure 5:**
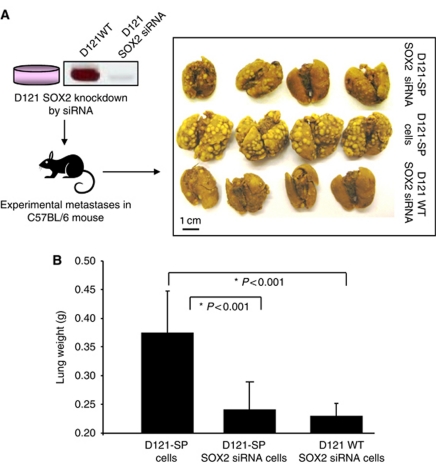
SOX2 knockdown in D121 lung carcinoma cells suppressed experimental pulmonary metastases in syngeneic C57/BL mice. (**A**) The data indicate the tumour foci on the lung surface of mice. (**B**) The lung weights of mice is shown by bar graphs.

To determine the effect of SOX2 knockdown in D121 cells on inhibition of tumour growth and metastases *in vivo*, C57BL/6 mice were injected i.v. with either 1 × 10^5^ D121-SP wild type or D121-SP SOX2 knockdown lung carcinoma cells. The data indicate that tumour foci on the lung surface of mice (Figure 5A) or lung weights (Figure 5B) of mice implanted with D121-SP SOX2 knock down cells are significantly smaller than that in mice implanted with wild-type D121-SP cells (*P*<0.001).

## Discussion

The cancer stem cell model of tumour development and progression implies that tumours contain a subset of cells that can both self-renew and give rise to a differentiated progeny (Eaves, 2008). As in other tissues, stem cells are a minority population of the whole organ, but are the only cells that can maintain tumour growth indefinitely. The remaining cells, though actively proliferating and making up the majority of cells in the tumour, are also differentiating and destined to die. Consequently, the self-renewal properties of CSCs are the real driving force behind tumour growth. Hence, the limited number of CSCs in the tumour and their specific phenotype are held responsible for tumour cell escape from conventional cancer therapies, resulting in disease relapse (Wegner, 1999; Graham *et al*, 2003). Thus, CSCs have been identified in a variety of solid malignancies with the identification of CSCs in these tumours being based on cell surface markers such as CD24, CD44, CD133 and Sca-1 (Singh *et al*, 1990; Al-Hajj *et al*, 2003; Xin *et al*, 2005). Cancer stem cells have been isolated by their ability to efflux the lipophilic Hoechst 33342 dye and are referred to as the ‘side population’.

In this study, we applied flow cytometry and the Hoechst 33342 dye efflux assay to isolate and characterize SP cells from the murine D121 non-small cell lung carcinoma cell line. Our data indicated SP cells to be enriched among D121 tumour cells when compared with non-SP cells. In general, the SP of D121 tumour cells is 1.8%, which displayed elevated expression of the ABCG2-ATP-binding cassette transporters on the cell surface, indicative of resistance to multiple chemotherapeutic drugs. Moreover, we found for the first time that SP isolated from D121 tumour cells express MHC-class I antigens when induced by exposure to IFN-*γ*; this might be of considerable importance for further eliciting T-cell-mediated immune response against such cancer stem cells, which could be critical for the development of effective cancer immunotherapy.

This new concept is strongly supported by the results of our study, indicating that the SP, isolated from murine D121 lung tumour cells by rapid Hoechst 33342 dye efflux, did exhibit resistance to chemotherapy. Indeed, RT–PCR and ICF further revealed an underlying molecular mechanism as the expression of SOX2 was markedly upregulated in SP isolated from D121 non-small lung cancer cells. The upregulation of such stem cell markers as ABCG2 and Sca-1 in the SP population isolated from D121 non-small cell lung cancer cells is also highly significant; especially as these makers are associated with multiple drug resistance. This finding correlates with the well-known function of chemoresistance by cancer stem cells, due in part to their state of dormancy in the G0/G1/phase of the cell cycle (Allan *et al*, 2006).

Our findings indicating that the SP population isolated from D121 non-small cell lung cancer cells reveals upregulation of well-known stem cell-associated transcription factors SOX2 and Oct 4. Correlates with recent findings by others that these two transcription factors form a heterodimeric molecular complex, which controls a transcriptional regulator network that helps to orchestrate the self-renewal and pluripotency of embryonic stem cells.

The siRNA-mediated knockdown of SOX2 in D121 lung carcinoma cells, which led to the decisive inhibition of these cells' migration in a transwell migration assay suggests that this transcription factor may regulate key biological functions of these cells. This contention was strengthened when we discovered by FACS analysis that apoptosis of SP cells was markedly increased by siRNA mediated knockdown of SOX2. Most importantly, we could demonstrate that the downregulated expression of SOX2 in SP achieved by siRNA knockdown did markedly decrease tumour growth and metastasis of D121 non-small cell lung cancer cells. In fact, the key role played by transcription factor SOX2 in this regard was clearly indicated when D121 lung cancer cells, subjected to SOX2 knock down *in vitro*, were subsequently injected i.v. into syngenic C57BL/6 mice. Together, our findings suggest that the SOX2 signalling pathway in CSCs derived from D121 lung cancer cells is activated and has a key role in the development and maintenance of cancer stem cells. Although the function of the SOX2 signalling pathway as well as its downstream genes *Oct 4* and *Nanog* in the development and maintenance of cancer stem cells is still being investigated, based on our experimental results, we propose that the SOX2 signaling pathway is involved in cancer stem cell development and that its deregulation can effectively suppress growth and metastasis of non-small cell lung carcinoma cells. This novel strategy may contribute to the future development of efficacious cancer treatments.
