# Downregulation of transcription factor SOX2 in cancer stem cells suppresses growth and metastasis of lung cancer

**DOI:** 10.1038/bjc.2011.210

**Published:** 2011-06-07

**Authors:** R Xiang, D Liao, T Cheng, H Zhou, Q Shi, T H Chuang, D Markowitz, R A Reisfeld, Y Luo

**Correction to:** *British Journal of Cancer* (2011) **104**, 1410–1417; doi:10.1038/bjc.2011.94

When published originally, earlier in Volume 104, the authors noticed that they had omitted to include TRDRP grant (19XT-0051) in the acknowledgements, which funded a large portion of the work for this paper. The authors are now happy to correct this omission.

